# Abstract of Address on the “Limitations of the Federal Food Law”

**Published:** 1913-11

**Authors:** Carl L. Alsberg

**Affiliations:** Chief Chemist, U. S. Department of Agriculture


### Abstract of Address on the "Limitations of the Federal Food Law."

BY DR. CARL L. ALSBERG, CHIEF CHEMIST, U. S. DEPARTMENT OF AGRICULTURE.

CHIEF OF BUREAU OF CHEMISTRY DISCUSSES RURAL SANITATION.

The States must protect the health of their citizens in food matters—transmission of diseases not limited to foods in Interstate Commerce—contends that protection of the people against organic infection is more important than regulation of misbranding.

Dr. Carl L. Alsberg, Chief of Bureau of Chemistry, U. S. Department of Agriculture, on September 9th delivered an address before the general session of the forty-first annual meeting of the American Public Health Association, then in session in Colorado Springs, Colo. Dr. Alsberg's subject related to the limitations of the Federal Food Law.

The following is an abstract of his remarks:

"Forty-eight States must rigidly inspect and control all easily contaminated foods and drugs produced within their borders. These include such foods as milk and other dairy products, water, fish, shell-fish, many meat products, candy, and, indeed, all food that is eaten raw or shipped exposed to the air. Each State owes this protection both to its own citizens and to those of other States who buy its products.

The individual State must guard the health of the State. The Federal authorities cannot do this work for the States. The Federal authority, under the Food and Drugs Act, extends only to food products entering into interstate commerce, and transmission of disease through infected food and water is not wholly an interstate matter. Disease frequently is of local source and local spread. The Food and Drugs Act is not an effective weapon against the spread within the State borders of disease-producing organisms in food. The Federal authorities cannot interfere with domestic commerce, but the State health officers are under no such limitations. Moreover, the Federal authorities have no right of entry and inspection into food factories within the State lines. A factory can be run under the most unsanitary conditions; milking may be done by a man recovering from scarlet fever, or milk may be produced on a farm where a member of the family is suffering from typhoid, and the Federal authorities have no power to act. Even if these products are shipped across the State line and samples are taken, there is no method for analysing a product which can supply evidence that the food is produced in unsanitary ways or within contaminating reach of epidemic or endemic diseases. The State authorities, however, can enter these factories, need not wait for shipment across State lines, and therefore, provided only that their laws are efficient and the funds at their disposal adequate, can prevent the sale of these deadly unlabeled foods.

FOOD AND DRUGS ACT LARGELY AN ECONOMIC MEASURE.

The Food and Drugs Act, as administered in the past has been very largely an economic measure. It has, to be sure, prevented very largely the mixing of active poisons in hurtful quantities with food products, but its particular work has been to see that food products are properly branded, so that the consumer knows what he is getting and is not cheated into paying a high price for a product adulterated with a cheaper. This must of course always be one of the purposes of the Act; but we must not be deceived into believing that this very important economic function of the Act is of great hygienic significance. Misbranding does not, demonstrably affect the death rate of the country. If the efforts devoted to prevention of misbranding were to be concentrated more largely upon the suppression of the traffic in contaminated milk, meat, vegetables and other products that may carry disease, a positive reduction of the country's death rate would inevitably result. At best, however, the Federal Authorities under the Food and Drugs Act can deal only with such of these bacteria-carrying products as chance to enter interstate commerce.

### FOOD AND DRUGS ACT USEFUL MAINLY TO BIG CITIES.

The Federal control of interstate commerce has in the past, and will have to be in the future, of more benefit to the large cities than to the small towns and rural communities, because the large cities even though in the center of the State draw their supplies to a far greater degree through interstate commerce than do the small towns and rural communities. The help that the Federal inspectors can extend to the rural communities, therefore, is absolutely limited to those settlements which happen to be near the borders of the State and so receive some of their easily contaminated foods through interstate commerce. The rural communities in the hearts of the States seldom obtain these unlabeled and possibly dangerous foods through interstate shipment. They get their food of this kind almost wholly in domestic commerce. Their milk comes from nearby farms. Their meats, if not slaughtered locally, come from nearby cities. Their fish and shell-fish may be shipped to them from sea-food centers in the nearest large towns within their State borders.

### 70,000,000 PEOPLE WITHOUT COMPLETE FOOD PROTECTION.

It is unfortunate that the large cities derive such preponderating advantages from the Federal control of interstate commerce. I say unfortunately advisedly, because the large cities need Federal protection far less than the small town or rural community. The reason is that systematic health protection work in the United States is largely confined to the big cities.

In many States the large cities are the only points which have any real sanitary protection. Our rural population of 49,000,000 people, including the 30,000,000 who live on farms, with the exception of those fortunate rural dwellers in one or two exceptional States which regard the health of the country people as also important, receive very little State health protection and maintain no local protective system of their own against contaminated local milk, meat, shell-fish, fish or vegetables. Moreover, only a small number of these rural inhabitants are safeguarded, by competent inspection, against polluted water supply or sewage disposal methods dangerous to health and life.

To this rural and unprotected population we can add the bulk of the 14,000,000 people who live in towns, cities and suburbs of less than 25,000 inhabitants, excepting those here and there who happen to dwell in the more progressive towns, or in those few hamlets located in the exceptional States that have already awakened to the importance of rural sanitation. If we wished we could go even further and add the population of many cities of 25,000 or 50,000 people which maintain no health officers; and of these cities which have health officers but so cripple them with poor laws or ordinances, or with insufficient funds, as to make their food inspection work most ineffective. I mean cities, for example, where boards of health are practically powerless under the law to seize milk except when it is actually watered or skimmed. These health officers must look on helplessly while milkmen send milk so dirty, or from such dangerous, disease-breeding places, as to make it a menace to the consumer. To prevent this deplorable condition it is absolutely necessary that uniform standards of bacteriological cleanliness as well as chemical composition be established. Bacteriological control is absolutely necessary to prevent the spread of contagion.

The probability, therefore, is that there are upward of 70,000,000 out of our 91,000,000 people who have no efficient and systematic protection from the major causes of the spread of typhoid, tuberculosis, deadly intestinal diseases of infants, scarlet fever, septic sore throat, trichinosis, and other ailments resulting from the circulation of disease producing organisms.

### LICENSE OF PHYSICIANS SHOULD BE CANCELLED FOR FAILURE TO REPORT CONTAGIOUS DISEASES.

We must not let ourselves be blinded by statistics, for the statistics of disease in the United States are notoriously unreliable.

For certain sections no attempt whatever is made to collect them. I need not tell an audience such as this that reliable statistics are the basis of all health protection. Until severe penalties are inflicted for failure to report cases of infectious diseases, until failure to do so results in the cancellation of the license to practice medicine there will be no really reliable statistics. And this will only be brought about when the State health services throughout the land furnish a permanent career for life instead of being, as they are but too often at present a side-line of politics—medical or otherwise. And these services should furnish a career like the Federal Civil Service, to sanitary engineers and chemists as well as to physicians. The people must learn that their health is too valuable to entrust to temporary health officers or to physicians who devote to the discharge of their duties as health officers, only such irregular moments as can be spared from their private practices.

RURAL DEATH STATISTICS INACCURATE.

The sections of the country in which as already stated little or no attention is paid to keeping records of disease are rural. The large cities keep some sort of record because attention to health protection and to easily contaminated foods is centralized almost wholly in the cities. With rare exceptions the effectiveness of the protective work depends on the individual city, rather than on a State-wide law or a State-wide and efficient health inspection system. Therefore, the collecting figures of urban deaths, look more significant, but if we could make an accurate total of the deaths on farms from the same causes, the figures would be appalling. In the country a man dies of "fever" or a "cough." The cities would announce that he died of "typhoid" or "tuberculosis." In the country babies fed on bad milk just die. In the city they die of accurately defined intestinal disorders and when the infantile death rate mounts, rigid milk inspection follows. In the cities, typhoid is often traced to its source and protective measures invoked. In the country the dangerous well or infected food supply usually continue to work havoc.

FOOD PROTECTION IN GREAT CITIES MAKES RURAL CONDITIONS WORSE.

One might suppose that where a city adopts and enforces a good health protection system, the educative effect of this must necessarily extend to the adjacent rural and suburban population. The truth is that an effective city health protective plan, where the surrounding rural districts have no adequate State or local protection, makes food conditions worse instead of better in the surrounding country districts. For example, Pittsburgh, Pa., and Cleveland, Ohio, have effective milk inspection. The health officer of one city notifies the health officer of the other city when milk entry has been refused to a certain dairy. This prevents that dairy from offering its refused milk to the other city. The results naturally would be that the producers of bad milk refused sale in these two larger cities could readily dispose of their product in the smaller places like McKees Rocks, Sewickley, Beaver Falls, and Coraopolis, which might have no effective milk inspection systems. The same thing is happening in many New Jersey towns. Milk refused entry into New York City, by the board of health is sent to towns like Perth Amboy, which may have health officers, but which do not provide them with funds enough for efficient milk inspection work and to carry on expensive prosecution.

DEPARTMENT CAN HELP RURAL COMMUNITIES NEAR STATE LINES.

The Department of Agriculture feels that it should give more attention to the protection of these rural communities. This means that the work hitherto largely confined to detection of the presence of preservatives in labeled foods which do not carry organic diseases, and the prosecutions for misbranding which might work a monetary fraud on the consumer, should be widely and rapidly extended to the control of interstate commerce in the dangerous unlabeled foods which can transmit and which do transmit serious diseases.

Plans for extending this work to interstate shipments of milk all over the country have already been made. It is the plan of the department to do more than exercise merely police control over interstate shipments. Plans have been made at the same time to show the milk producer how to produce better milk; and also to show him that it will pay him to produce his milk under the best conditions. The educational and the regulatory work will go on together—an ancient combination much used by the old fashioned school teacher who taught by precept when he could and resorted to the switch when he had to.

The only help that the department's work in the rural communities around the State's edges can be to the country districts in the heart of the State is to educate, through example and by means of publicity, the rural population throughout all the States to demand of the State that it extend to them the same protection now enjoyed by the large cities against preventable disease disseminated through their food. It is along these lines that the State officials can co-operate with the Federal government. They can help to arouse the public to demand effective State-wide measures in all the States, and to insist on an efficient, permanent staff of health officials, and rigorous supervision of the preparation of all foods liable to contamination and pollution. The Department of Agriculture will do its duty not merely in exercising control over interstate commerce, but also in helping food producers bring their food up to proper quality, and it will thus add materially to the available supply of honest and safe food in the country. The great purpose of the Department of Agriculture, is a constructive one, namely, not merely to punish adulterators of food, but to help honest manufacturers to discharge their duty to the community by supplying wholesome products. The actual detection and closing of dirty or unsanitary factories and dairies, or the compelling of their owners to mend their methods, must, however, rest with the States.

---

No Babies.—At the wedding reception the young man remarked: "Wasn't it annoying the way that baby cried during the whole ceremony?"

"It was simply dreadful," replied the prim little maid of honor, "and when I get married I'm going to have engraved right in the corner of the invitations: 'No babies expected.' "

---

The Sign of a Frontier Quack.—Legs and arms sawed off while you wait without pain. Child-birth and tumore a specialty.

No odds asked in measles, hooping-coff, mumps or diarar.

Bald-head, bunions, corns or warts, cancer, ingrowing tow-nails treated scientifically.

Coleck, cramps, costiveness nailed on sight.

Wring-worms, pole-evil, shingles, moles and cross-eye cured in one treatment or no pay.

P. S.—Terms: Cash invariably in advance. No cure no pay.

N. B. (Take notis) No coroner never yet sot on the remanes of my customers, and enny one hiring me don't hafto be layin up money to buy a gravestone. Come on come all.—*Exchange.*

